# Characteristics of suicide among farmers and ranchers: Using the CDC NVDRS 2003–2018

**DOI:** 10.1002/ajim.23399

**Published:** 2022-06-07

**Authors:** Cristina D. M. Miller, Josie M. Rudolphi

**Affiliations:** ^1^ United States Department of Agriculture Washington, DC USA; ^2^ Rural Development Innovation Center, Data Analytics Division University of Illinois Urbana‐Champaign Urbana Illinois USA

**Keywords:** depression, farmer, farmer suicide, physical health problems, rancher, suicide

## Abstract

**Background:**

Suicide is among the top 10 causes of premature death in the United States. This study provides details on farmer and rancher suicide decedents, including demographic information, mental health status, history of suicidal thoughts and attempts, and circumstances associated with death.

**Methods:**

Data for this study were obtained from the Centers for Disease Control and Prevention's National Violent Death Reporting System Restricted Access Database for the years 2003–2018. Descriptive statistics and adjusted odds ratios are presented for farm and nonfarm populations in addition to farm populations by age groups and sex.

**Results:**

This study found that almost half of the farmer suicide decedents were over 65 years old. Firearms were the most widely used method for farmers and ranchers regardless of age and sex. Young farmers and ranchers that died by suicide were more likely to have had relationship problems and older farmers and ranchers that died by suicides were more likely to have had a physical health problem. Male farmer and rancher suicide decedents were more likely to die by firearm than females, and female farmer and rancher suicide decedents were likely to have resided in a small metropolitan area, however, due to small numbers and suppression in the data, most sex comparisons were not able to be presented.

**Conclusions:**

While no clear risk factor for suicide among farmers and ranchers emerged, results underscore the complex nature of suicide and the need for multifaceted, culturally competent interventions and campaigns that address suicide risk and prevention at the individual and community levels.

## 1 INTRODUCTION

Suicide is ranked among the top 10 leading causes of premature death in the United States.^1^ In 2019, the US suicide rate was 14.5 per 100,000 people,^2^ a rate that has increased over 30% since 1999.^3^ However, this rate masks a serious public health crisis among males, especially those over the age of 20 living in rural America. In 2019, the suicide rate for males over the age of 20 was 29.4 per 100,000,^4^ but when examining suicide rates across levels of rurality a worsening pattern appears. For males over the age of 20 in 2019, the metropolitan suicide rate was 27.7 per 100,000 (a rate increase of 26.7% since 1999); the micropolitan suicide rate was 37.8 per 100,000 (a rate increase of 36.8% since 1999); the rural noncore suicide was 40.4 per 100,000 (a rate increase of 39.4% since 1999).^4^ These increasing suicide rates among males ages 20 and older indicate a serious issue and reflects the difficulty that suicide awareness and prevention efforts have in identifying and reaching people who are at risk.^5^

As part of any suicide prevention strategy, understanding the risk factors associated with suicide are vitally important.^6^ Risk factors for suicide in the United States include demographic, residential, and circumstance characteristics. The loss of a job or events causing major fluctuations in household income; or life events such as death of a family member or friend, divorce or separation, conflict or argument with a spouse or relative; or a serious physical health problem or mental health problem are examples of risk factors that may be possible pathways to depressive mental illness and potentially may lead to a suicide attempt.^6^, ^7^ Additionally, community and societal risk factors include rurality, access and barriers to health care, access to lethal means, and stigma associated with mental illness contribute to suicide risk.^8^, ^9^ Evidence suggests some occupations are at increased risk of suicide when compared to others, suggesting there are occupational risk factors as well.^10^, ^11^

Farmers and ranchers may be at increased risk of suicide when considering demographic, environmental, and occupational circumstances common to population. In the United States, the average age of principal operators—farmers and ranchers—is 58.6 years and more than 1.94 million are male, or 71%.^12^ Farmers and ranchers commonly report health conditions such as chronic pain, hearing loss, and/or permanent disability which may also increase risk of suicide.^13^, ^14^ Individuals engaged in agricultural production experience unique, occupational stressors and negative life events that threaten their mental health, including inconsistent work schedules, fluctuating commodity prices, shrinking labor pools, uncertain government policies, and unpredictable environmental conditions.^15^, ^16^, ^17^, ^18^, ^19^

State‐specific analyses, while becoming dated, have suggested farmers die by suicide at higher rates than individuals in other occupations.^20^, ^21^ In the latest Center for Disease Control and Prevention (CDC) report on suicides by industry and occupation, using data from 2016 (including only 17 states and individuals in the labor force aged 16–64), the agriculture, forestry, fishing, and hunting *industry* sector has the fourth highest suicide rate among males and had an increased rate of suicide when compared to all males in the study population (36.1 per 100,000 vs. 27.4 per 100,000).^11^ That same report (including only 17 states and workers aged 16–64), ranks the *occupation* of farmers, ranchers, and other agriculture managers fourth in male suicides (31.4 per 100,000).^10^, ^11^

However, previous analyses of farmer suicides have included retired farmers, agricultural workers, fishers, loggers, and agricultural service personnel^22^, ^23^, ^24^ and it is important to be cognizant that fishers, loggers, and nonproduction occupations (such as farm laborers) experiences may differ considerably from those of an agricultural producer, or farmer. An analysis that considers farmers separate from fishers, foresters, hunters, and nonproduction occupations may identify specific risk factors for suicide and inform appropriate and relevant suicide prevention programming and interventions. Many farmers and ranchers work and reside in rural areas, where rates of suicide are increasing^8^, ^9^ and lacking in mental health professionals.^25^ In 2018, 79% of rural‐nonadjacent counties (nonmetropolitan counties that are not adjacent to metropolitan or micropolitan counties) had no psychiatrists.^26^

The objective of this study is to identify potential suicide risk factors and compare farmer and rancher suicide decedents across ages (age 18–45, 46–64, and 65 and older), sex (male and female), rurality, as well as to the nonfarmer suicide decedents in the data. We hypothesize that certain stressors may have more of an impact on individuals at different life stages, We also hypothesize that female farmers may have reached out to mental health professionals and family or friends about their mental state more than male farmers, although the stressors might be the roughly the same due to the pressures of the occupation, in general.

## 2 MATERIALS AND METHODS

### 2.1 Data source and study population

Data for this study were obtained from the CDC National Violent Death Reporting System (NVDRS) Restricted Access Database from 2003 to 2018. As a state‐based surveillance system, NVDRS collects mortality data from death certificates and combines the data with additional information from coroner and medical examiner records as well as law enforcement agency reports to better understand the circumstances and characteristics associated with violence‐related deaths—such as homicides, suicides, and unintentional firearm deaths—in participating states and territories.^27^, ^28^

The data set originated in 2003 with only seven states reporting data to NVDRS. These states included Alaska, Maryland, Massachusetts, New Jersey, Oregon, South Carolina, and Virginia. By 2018, 40 states reported data, although some of those states are still not fully reporting. The states reporting in 2018 included Alabama, Alaska, Arizona, California, Colorado, Connecticut, Delaware, Georgia, Hawaii, Illinois, Indiana, Iowa, Kansas, Kentucky, Louisiana, Maine, Maryland, Massachusetts, Michigan, Minnesota, Missouri, Nebraska, Nevada, New Hampshire, New Jersey, New Mexico, New York, North Carolina, Ohio, Oklahoma, Oregon, Pennsylvania, Rhode Island, South Carolina, Utah, Vermont, Virginia, Washington, West Virginia, and Wisconsin. We pooled the NVDRS data from 2003 to 2018. CDC does not require Institutional Review Board review for this study.

The CDC National Institute for Occupational Safety and Health (NIOSH) recently enhanced the data by introducing a set of standardized industry and occupation codes, such as the codes from Census. To identify farmers and ranchers, we limit our analysis to suicide decedents, 18 years of age and older, who met the following industry classifications—*Crop production* (Census Code 0170) or *Animal production* (Census Code 0180), and the following occupation classification—*Farmers, ranchers, and other agricultural managers* (Census Code 0205). We did not restrict farmers and ranchers by geography (urban/rural).

Our study consists of 1363 crop producers and 572 animal producers in the data combining to a total of 1935 crop producers and animal producers, hereafter referred to as farmers and ranchers. Within farmer and rancher suicide decedents, 97 are female and 1838 are male. For nonfarmers, we limit our analysis to suicide decedents 18 years of age or older. We did not restrict nonfarmers by geography (urban/rural), industry, or employment status (such as employed, student, or homemaker). The number of nonfarmers who died by suicide in our sample totaled 223,349. In the age group analysis, the counts by age group are: age 18–45: 455 farmers and 105,419 nonfarmers; age 46–64: 619 farmers and 80,556 nonfarmers; age 65 and older: 861 farmers and 37,238 nonfarmers. These counts vary slightly in the tables due to missing data.

We use the United States Department of Agriculture Economic Research Service Urban Influence Codes (UIC) to understand the geographical location of the suicide decedents. Using the UIC codes, which are based on the Office of Management and Budget definitions for metropolitan and micropolitan areas, we classify the counties by five categories: large metropolitan (counties with an urban area of 1+ million residents), small metropolitan (counties with an urban area of less than 1 million residents but more than 50,000), micropolitan (counties with an urban area of 10,000–49,999 residents), rural noncore (counties with an urban area of less than 10,000 residents) adjacent to metropolitan or micropolitan county, and rural noncore (counties with an urban area of less than 10,000 residents) not adjacent to metropolitan or micropolitan county (considered the most remote rural areas).^29^

### 2.2 Variables

Aside from the descriptive statistics of the suicide decedent, we include statistics that may provide insight into the decision‐making process of the suicide decedent, such as the mechanism used in the suicide, whether there was any evidence of mental health or substance use problems, and potential life stressors or interpersonal problems surrounding the event. Table S1 provides a description of all of the variables that we included.

#### 2.2.1 Mechanism used for death by suicide

The NVDRS data provides several means of death by suicide which include *firearm, hanging (including strangulation or suffocation), poisoning, drowning, falls, fire/burns, sharp instrument, motor vehicle (including buses or motorcycles), other (such as taser, electrocution, nail gun), nonpowder gun, or other transport vehicles (such as trains, planes, boats)*. We report results of farmer and nonfarmer populations who died by suicide using the following categories: firearms, hanging, poisoning, or other methods. We classify the following means of death as “other methods” due to thinness in the data—drowning, falls, fire/burns, sharp instrument, motor vehicle including buses or motorcycles, other (such as taser, electrocution, nail gun), nonpowder gun, other transport vehicles (such as trains, planes, boats).

#### 2.2.2 Mental health/substance use problems and event history

The following variables are included in our analysis and may provide insight into the individual's mental health status and substance use problems, including mental health problems, previously received mental health treatment, depressed mood, known alcohol problems or substance abuse problems, and history of suicidal thought or suicidal attempts.

#### 2.2.3 Life stressors and interpersonal situations

Potential life stressors or interpersonal situations that may have influenced the decision‐making process of the individual to die by suicide include financial problems, physical health problems, job problems, death of a friend or family member, recent suicide of family or friend, recent eviction or loss of home, recent criminal legal problems, intimate partner problems, family relationship problems, or an argument or conflict.

### 2.3 Data analysis

In this study, we present descriptive statistics and adjusted odds ratios (aORs) for farmer and nonfarmer populations. Within the farmer population, we also present statistics and aORs for farmers by sex and age group. We report counts and percentages as well as across‐group *p*‐values based on Pearson's *χ* ^2^ indicating the level of confidence (*p* < 0.05) that the percentages are independent across groups in the sample. Due to the conditions of a data use agreement with CDC, we suppress the descriptive statistics results if the count is below a minimum threshold. In addition, due to the conditions of a data use agreement with CDC, we suppress the aORs if the counts are below a minimum threshold that would affect the statistical reliability of the results. In the tables, where suppression is necessary, we insert an *s* in the cell. In the aORs, where the results are not statistically reliable, we insert NSR (not statistically reliable).

## 3 RESULTS

The demographic and incident characteristics from Tables 1 to 4 are presented by farmers compared to nonfarmers, female farmers compared to male farmers, and then farmers compared to nonfarmers by age groups (18–45, 46–64, and 65 and older).

**Table 1:** Demographic characteristics of suicide decedents, 40 states,^a^ 2003–2018

	Farmer/rancher, *N* (%)	Nonfarmer/rancher, *N* (%)	aOR^b^ (95% CI)	Male farmer/rancher, *N* (%)	Female farmer/rancher, *N* (%)	aOR^c^ (95% CI)
Age^a^ (mean in years, SD)^d^	60.2 (±0.4)	47.2 (±0.04)	–	60.6 (±0.5)	52.9 (±1.7)	–
Age range^a^						
18–45^d^	455 (23.5)	105,419 (47.2)	–	425 (23.1)	30 (30.9)	–
46–64^d^^,^ e	619 (32.0)	80,556 (36.1)	–	574 (31.2)	45 (46.4)	–
65+^d^^,^ e	861 (44.5)	37,238 (16.7)	–	839 (45.7)	22 (22.7)	–
Gender^b^						
Male^d^	1838 (95.0)	173,811 (77.8)	–	–	–	–
Female^d^	97 (5.0)	48,538 (22.2)	–	–	–	–
Race/ethnicity^b^						
White^d^	1760 (91.0)	187,170 (83.8)	–	1669 (90.8)	91 (93.8)	–
Black/two races/other^d^	22 (1.1)	14,007 (6.3)	–	22 (1.2)	0 (0.0)	–
American Indian/Alaska Native	29 (1.5)	2569 (1.2)	–	29 (1.6)	0 (0.0)	–
Asian/Pacific Islander	41 (2.1)	4592 (2.1)	–	37 (2.0)	s	–
Hispanic/LatinX^d^	67 (3.5)	11,719 (5.3)	–	66 (3.6)	s	–
Marital status^b^						
Single/never married^d^^,^ e	471 (24.3)	75,185 (33.7)	1.1** (1.0–1.3)	456 (24.8)	15 (15.5)	NSR
Married^d^	837 (43.3)	76,741 (34.4)	1.1** (1.0–1.2)	801 (43.6)	36 (37.1)	0.9 (0.6–1.4)
Divorced/separated^d^	352 (18.2)	55,267 (24.7)	0.7*** (0.6–0.8)	328 (17.9)	24 (24.7)	1.5 (0.9–2.4)
Widowed^d^^,^ e	263 (13.6)	13,446 (6.0)	1.3*** (1.1–1.5)	241 (13.1)	22 (22.7)	4.2*** (2.3–7.7)
Veteran status						
Veteran^d^^,^ e	427 (22.4)	40,065 (19.2)	0.5*** (0.5–0.6)	424 (23.5)	s	NSR
Education^d^						
Less than high school diploma^d^^,^ e	240 (32.4)	12,833 (14.4)	2.5*** (2.1–2.9)	237 (33.3)	s	NSR
High school graduate	329 (44.4)	37,985 (42.6)	1.1* (1.0–1.3)	313 (44.0)	16 (53.3)	NSR
Some college^d^	114 (15.4)	19,657 (22.0)	0.8** (0.6–1.0)	108 (15.2)	6 (20.0)	NSR
College graduate and above^d^	58 (7.8)	18,734 (21.0)	0.3*** (0.2–0.4)	53 (7.5)	5 (16.7)	NSR
Location^b^						
Large metropolitan^d^	294 (15.2)	102,537 (45.9)	0.2*** (0.2–0.3)	275 (15.0)	19 (19.6)	NSR
Small metropolitan^d^^,^ e	526 (27.2)	77,636 (34.8)	0.7*** (0.6–0.8)	486 (26.4)	40 (41.2)	1.9*** (1.2–2.9)
Micropolitan^d^	466 (24.1)	25,340 (11.4)	2.3*** (2.0–2.5)	447 (24.3)	19 (19.6)	NSR
Rural, adjacent to metro/micro^d^^,^ e	514 (26.6)	14,073 (6.3)	4.8*** (4.3–5.3)	499 (27.2)	15 (15.5)	NSR
Rural, not adjacent to metro/micro	135 (7.0)	3763 (1.7)	3.8*** (3.1–4.6)	131 (7.1)	s	NSR

*Note*: All race groups are non‐Hispanic.

Alphabet superscripts indicate the population counts, which vary due to missing data. Variables with superscript: (a) Farmer *N* = 1935, nonfarmer *N* = 223,213, male *N* = 1838, female *N* = 97. (b) Farmer *N* = 1935, nonfarmer *N* = 223,349, male *N* = 1838, female *N* = 97. (c) Farmer *N* = 1903, nonfarmer *N* = 209,132, male farmer *N* = 1808, female farmer *N* = 95. (d) Farmer *N* = 741, nonfarmer *N* = 89,209, male farmer *N* = 711, female farmer *N* = 30.

s Indicates that the value had to be suppressed, due to a low cell count.

Abbreviations: aOR, adjusted odds ratio; CI, confidence interval.

^a^ Alabama, Alaska, Arizona, California, Colorado, Connecticut, Delaware, Georgia, Hawaii, Illinois, Indiana, Iowa, Kansas, Kentucky, Louisiana, Maine, Maryland, Massachusetts, Michigan, Minnesota, Missouri, Nebraska, Nevada, New Hampshire, New Jersey, New Mexico, New York, North Carolina, Ohio, Oklahoma, Oregon, Pennsylvania, Rhode Island, South Carolina, Utah, Vermont, Virginia, Washington, West Virginia, and Wisconsin (not all states are fully reporting).

^b^ Adjusted odds ratios measure the association between the decedent having the demographic characteristic and being a farmer/rancher. Each adjusted odds ratio used nonfarmer as the reference group and controlled for age, race/ethnicity, and sex. Therefore, odd ratios for age groups, race/ethnicity, and sex are not presented. Adjusted odds ratio level of statistical significance is indicated at *p < 0.10, **p < 0.05, ***p < 0.01. NSR = not statistically reliable. Statistical reliability criteria for logistic regression not met because cell frequencies were less than the required minimum.

^c^ Adjusted odds ratios measure the association between the decedent having the demographic characteristic and being a female farmer/rancher. Each adjusted odds ratio used male farmer/rancher as the reference group and controlled for age and race/ethnicity. Thus, odds ratios for age groups, race/ethnicity, and sex are not presented. Adjusted odds ratio level of statistical significance is indicated at *p < 0.10, **p < 0.05, ***p < 0.01. NSR = not statistically reliable. Statistical reliability criteria for logistic regression not met because cell frequencies were less than the required minimum.

^d^ Pearson's *χ* ^2^ test result for difference between farmer/rancher and nonfarmer/rancher is significant at *p* < 0.05.

^e^ Pearson's *χ* ^2^ test result for difference between female farmer and male farmer is significant at *p* < 0.05.

*Source*: CDC National Violent Death Reporting System data from reporting states (2003–2018).

**Table 2:** Incident characteristics of suicide decedents, 40 states,^a^ 2003–2018

	Farmer/rancher, *N* (%)	Nonfarmer/rancher, *N* (%)	aOR^b^ (95% CI)	Male farmer/rancher, *N* (%)	Female farmer/rancher, *N* (%)	aOR^c^ (95% CI)
Mechanism						
Firearm^d^^,^ e	1436 (74.2)	112,249 (50.3)	1.9*** (1.7–2.1)	1391 (75.7)	45 (46.4)	0.3*** (0.2–0.4)
Hanging^d^^,^ e	312 (16.1)	57,718 (25.8)	0.7*** (0.7–0.8)	288 (15.7)	24 (24.7)	1.7** (1.0–2.8)
Poisoning^d^^,^ e	108 (5.6)	35,238 (15.8)	0.5*** (0.4–0.6)	90 (4.9)	18 (18.6)	NSR
Other^d^^,^ e	79 (4.1)	18,144 (8.1)	0.6*** (0.4–0.7)	69 (3.8)	10 (10.3)	NSR
Event history						
History of suicidal thoughts^d^^,^ e	298 (15.4)	43,786 (19.6)	0.8*** (0.7–0.9)	275 (15.0)	23 (23.7)	1.6* (1.0–2.7)
History of suicide attempts^d^^,^ e	149 (7.7)	40,543 (18.2)	0.6*** (0.5–0.7)	128 (7.0)	21 (21.7)	3.0*** (1.8–5.1)
Life stressors						
Financial problems	162 (8.4)	21,360 (9.6)	0.9 (0.8–1.1)	150 (8.2)	12 (12.4)	NSR
Physical health problems^d^	631 (32.6)	44,679 (20.0)	1.0 (0.9–1.2)	602 (32.8)	29 (29.9)	1.3 (0.8–2.2)
Job problems^d^	111 (5.7)	23,451 (10.5)	0.6*** (0.5–0.8)	106 (5.8)	5 (5.2)	NSR
Death of a friend or family^d^	143 (7.4)	13,096 (5.9)	1.1 (0.9–1.3)	135 (7.3)	8 (8.3)	NSR
Recent criminal legal problem^d^	116 (6.0)	18,276 (8.2)	0.9 (0.7–1.1)	114 (6.2)	s	NSR
Recent suicide of friend or family	35 (1.8)	4161 (1.9)	1.1 (0.7–1.5)	31 (1.7)	s	NSR
Eviction or loss of home^d^	32 (1.7)	6654 (3.0)	0.6*** (0.4–0.8)	29 (1.6)	s	NSR
Interpersonal						
Intimate partner problem^d^	320 (16.5)	58,971 (26.4)	0.8*** (0.7–0.9)	299 (16.3)	21 (21.7)	1.1 (0.7–1.8)
Family relationship problem^d^	97 (5.0)	14,395 (6.5)	0.9 (0.8–1.1)	89 (4.6)	8 (8.3)	NSR
Argument or conflict^d^^,^ e	118 (6.1)	22,881 (10.2)	0.8*** (0.6–0.9)	106 (5.8)	12 (12.4)	NSR
Mental health/substance use						
Mental health problems^d^^,^ e	613 (31.7)	95,229 (42.6)	0.8*** (0.7–0.9)	567 (30.9)	46 (47.4)	1.8*** (1.2–2.8)
Received mental health treatment^d^^,^ e	367 (19.0)	60,170 (26.9)	0.8*** (0.7–0.9)	332 (18.1)	35 (36.1)	2.4*** (1.5–3.7)
Depressed mood	689 (35.6)	77,566 (34.7)	1.0 (0.9–1.1)	652 (35.5)	37 (38.1)	1.1 (0.7–1.7)
Alcohol problem^d^	217 (11.2)	37,235 (16.7)	0.7*** (0.6–0.8)	207 (11.3)	10 (10.3)	NSR
Substance abuse problem^d^	119 (6.2)	32,835 (14.7)	0.6*** (0.5–0.7)	115 (6.3)	s	NSR

*Note*: Population counts: Farmer *N* = 1935, nonfarmer *N* = 223,349, male *N* = 1838, female *N* = 97.

s Indicates that the value had to be suppressed, due to a low cell count.

Abbreviations: aOR, adjusted odds ratio; CI, confidence interval.

^a^ Alabama, Alaska, Arizona, California, Colorado, Connecticut, Delaware, Georgia, Hawaii, Illinois, Indiana, Iowa, Kansas, Kentucky, Louisiana, Maine, Maryland, Massachusetts, Michigan, Minnesota, Missouri, Nebraska, Nevada, New Hampshire, New Jersey, New Mexico, New York, North Carolina, Ohio, Oklahoma, Oregon, Pennsylvania, Rhode Island, South Carolina, Utah, Vermont, Virginia, Washington, West Virginia, and Wisconsin (not all states are fully reporting).

^b^ Adjusted odds ratios measure the association between the decedent having the incident characteristic and being a farmer/rancher. Each adjusted odds ratio used nonfarmer as the reference group and controlled for age, race/ethnicity, and sex. Adjusted odds ratio level of statistical significance is indicated at *p < 0.10, **p < 0.05, ***p < 0.01. NSR = not statistically reliable. Statistical reliability criteria for logistic regression not met because cell frequencies were less than the required minimum.

^c^ Adjusted odds ratios measure the association between the decedent having the incident characteristic and being a female farmer/rancher. Each adjusted odds ratio used male farmer/rancher as the reference group and controlled for age and race/ethnicity. Adjusted odds ratio level of statistical significance is indicated at *p < 0.10, **p < 0.05, ***p < 0.01. NSR = not statistically reliable. Statistical reliability criteria for logistic regression not met because cell frequencies were less than the required minimum.

^d^ Pearson's *χ* ^2^ test result for difference between farmer/rancher and nonfarmer is significant at *p* < 0.05.

^e^ Pearson's *χ* ^2^ test result for difference between female farmer and male farmer is significant at *p* < 0.05.

*Source*: CDC National Violent Death Reporting System data from reporting states (2003–2018).

**Table 3:** Demographic characteristics of suicide decedents by age, 40 states,^a^ 2003–2018

	Age 18‐45			Age 46‐64			Age 65 and over		
Mechanism	Farmer/rancher, *N* (%)	Nonfarmer/rancher, *N* (%)	aOR^b^ (95% CI)	Farmer/rancher, *N* (%)	Nonfarmer/rancher, *N* (%)	aOR^c^ (95% CI)	Farmer/rancher, *N* (%)	Nonfarmer/rancher, *N* (%)	aOR^d^ (95% CI)
Gender^f^									
Male^e^^,^ f ^,^ g	425 (93.4)	82,884 (78.62)	–	574 (92.7)	60,016 (74.5)	–	839 (97.4)	30,781 (82.7)	–
Female^e^^,^ ^f^ ^,^ g	30 (6.6)	22,528 (21.4)	–	45 (7.3)	20,537 (25.5)	–	22 (2.6)	6456 (17.3)	–
Race/ethnicity^f^									
White^e^^,^ f	376 (82.6)	80,627 (76.5)	–	580 (93.7)	71,975 (89.4)	–	804 (93.4)	34,490 (92.6)	–
Black/two races/other^e^^,^ ^f^ ^,^ g	6 (1.3)	9687 (9.2)	–	6 (1.0)	3286 (4.1)	–	10 (1.2)	1029 (2.8)	–
American Indian/Alaska Native^e^	18 (4.0)	1972 (1.9)	–	6 (1.0)	499 (0.6)	–	5 (0.6)	98 (0.3)	–
Asian/Pacific Islander^g^	7 (1.5)	2759 (2.6)	–	10 (1.6)	1235 (1.5)	–	24 (2.8)	596 (1.6)	–
Hispanic/LatinX	39 (8.6)	8276 (7.9)	–	14 (2.3)	2690 (3.3)	–	14 (1.6)	750 (2.0)	–
Marital status^f^									
Single/never married^g^	260 (57.1)	58,386 (55.4)	1.2 (0.9–1.4)	109 (17.6)	14,216 (17.7)	1.0 (0.8–1.2)	102 (11.9)	2567 (6.9)	2.1*** (1.7–2.6)
Married^f^	125 (27.5)	26,431 (25.1)	1.1 (0.9–1.4)	300 (48.5)	32,983 (40.9)	1.3*** (1.1–1.5)	412 (47.9)	17,293 (46.4)	1.0 (0.9–1.2)
Divorced/separated^e^^,^ ^f^ ^,^ g	58 (12.8)	18,599 (17.6)	0.6*** (0.5–0.8)	181 (29.2)	28,620 (35.5)	0.8*** (0.6–0.9)	113 (13.1)	8023 (21.6)	0.7*** (0.5–0.8)
Widowed	7 (1.5)	876 (0.8)	NSR	27 (4.4)	3627 (4.5)	1.1 (0.7–1.6)	229 (26.6)	8940 (24.0)	0.9 (0.8–1.1)
Veteran status									
Veteran^e^^,^ ^f^ ^,^ g	30 (6.7)	10,449 (10.6)	0.5*** (0.4–0.8)	61 (10.0)	12,985 (17.3)	0.4*** (0.3–0.5)	336 (39.9)	16,607 (47.2)	0.4*** (0.4–0.5)
Education^h^									
Less than high school diploma^e^^,^ ^f^ ^,^ g	30 (20.8)	6334 (15)	1.4* (1.0–2.1)	50 (20.5)	3662 (11.4)	2.0*** (1.4–2.7)	160 (45.3)	2833 (19.4)	3.1*** (2.5–3.9)
High school graduate^e^	77 (53.5)	19,116 (45.1)	1.3 (0.9–1.8)	115 (47.1)	13,249 (41.2)	1.2* (1.0–1.6)	137 (38.8)	5612 (38.4)	1.0 (0.8–1.3)
Some college^g^	26 (18.1)	10,218 (24.1)	0.8 (0.5–1.2)	54 (22.1)	7023 (21.8)	1.1 (0.8–1.5)	34 (9.6)	2415 (16.5)	0.6*** (0.4–0.9)
College graduate and above^e^^,^ ^f^ ^,^ g	11 (7.6)	6702 (15.8)	NSR	25 (10.3)	8265 (25.7)	0.3*** (0.2–0.5)	22 (6.2)	3767 (25.8)	0.2*** (0.1–0.3)
Location^f^									
Large metropolitan^e^^,^ ^f^ ^,^ g	70 (15.4)	48,895 (46.4)	0.2*** (0.2–0.3)	93 (15.0)	37,802 (46.9)	0.2*** (0.2–0.3)	131 (15.2)	15,791 (42.4)	0.2*** (0.2–0.3)
Small metropolitan^f^^,^ g	148 (32.5)	36,981 (35.1)	0.9 (0.7–1.0)	153 (24.7)	27,579 (34.2)	0.6*** (0.5–0.7)	225 (26.1)	13,041 (35.0)	0.7*** (0.6–0.8)
Micropolitan^e^^,^ ^f^ ^,^ g	97 (21.3)	11,736 (11.1)	2.0*** (1.6–2.5)	146 (23.6)	8780 (10.9)	2.4*** (2.0–2.9)	223 (25.9)	4795 (12.9)	2.3*** (2.0–2.7)
Rural, adjacent to metro/micro^e^^,^ ^f^ ^,^ g	105 (23.1)	6028 (5.7)	4.6*** (3.7–5.8)	180 (29.1)	5160 (6.4)	5.8*** (4.9–6.9)	229 (26.6)	2866 (7.7)	4.3*** (3.6–5.0)
Rural, not adjacent to metro/micro^e^^,^ ^f^ ^,^ g	35 (7.7)	1779 (1.7)	4.2*** (2.8–6.3)	47 (7.6)	1235 (1.5)	5.1*** (3.7–6.9)	53 (6.2)	745 (2.0)	2.9*** (2.2–3.9)

*Note*: All race groups are non‐Hispanic.

Alphabet superscripts indicate the population counts, which vary due to missing data. Variables with superscript: (f) Age 18–45: Farmer *N* = 455, nonfarmer *N* = 105,419; age 46–64: Farmer *N* = 619, nonfarmer *N *= 80,556; age 65p: Farmer *N* = 861, nonfarmer *N* = 37,238. (g) Age 18–45: Farmer *N* = 447, nonfarmer *N* = 98,876; age 46–64: Farmer *N* = 613, nonfarmer *N* = 75,015; age 65p: Farmer *N* = 843, nonfarmer *N* = 35,165. (h) Age 18–45: Farmer *N* = 144, nonfarmer *N* = 42,370; age 46–64: Farmer *N* = 244, nonfarmer *N* = 32,199; age 65p: Farmer *N* = 353, nonfarmer *N* = 14,627.

s Indicates that the value had to be suppressed, due to a low cell count.

Abbreviations: aOR, adjusted odds ratio; CI, confidence interval.

^a^ Alabama, Alaska, Arizona, California, Colorado, Connecticut, Delaware, Georgia, Hawaii, Illinois, Indiana, Iowa, Kansas, Kentucky, Louisiana, Maine, Maryland, Massachusetts, Michigan, Minnesota, Missouri, Nebraska, Nevada, New Hampshire, New Jersey, New Mexico, New York, North Carolina, Ohio, Oklahoma, Oregon, Pennsylvania, Rhode Island, South Carolina, Utah, Vermont, Virginia, Washington, West Virginia, and Wisconsin (not all states are fully reporting).

^b^ Adjusted odds ratios measure the association between the decedent having the demographic characteristic and being a farmer/rancher aged 18–45. The adjusted odds ratio used nonfarmer age 18–45 as the reference group and controlled for race/ethnicity and sex. Therefore, odd ratios for age, race/ethnicity, and sex are not presented. Adjusted odds ratio level of statistical significance is indicated at *p < 0.10, **p < 0.05, ***p < 0.01. NSR = not statistically reliable. Statistical reliability criteria for logistic regression not met because cell frequencies were less than the required minimum.

^c^ Adjusted odds ratios measure the association between the decedent having the demographic characteristic and being a farmer/rancher aged 46–64. The adjusted odds ratio used nonfarmer age 46–64 as the reference group and controlled for race/ethnicity and sex. Thus, odds ratios for age, race/ethnicity, and sex are not presented. Adjusted odds ratio level of statistical significance is indicated at *p < 0.10, **p < 0.05, ***p < 0.01. NSR = not statistically reliable. Statistical reliability criteria for logistic regression not met because cell frequencies were less than the required minimum.

^d^ Adjusted odds ratios measure the association between the decedent having the demographic characteristic and being a farmer/rancher aged 65 and older. Each adjusted odds ratio used nonfarmer age 65 and older as the reference group and controlled for race/ethnicity and sex. Thus, odds ratios for age, race/ethnicity, and sex are not presented. Adjusted odds ratio level of statistical significance is indicated at *p < 0.10, **p < 0.05, ***p < 0.01. NSR = not statistically reliable. Statistical reliability criteria for logistic regression not met because cell frequencies were less than the required minimum.

^e^ Pearson's *χ* ^2^ test result for difference between farmer/rancher and nonfarmer/rancher age 18–45 is significant at *p* < 0.05.

^f^ Pearson's *χ* ^2^ test result for difference between farmer/rancher and nonfarmer/rancher age 46–64 is significant at *p* < 0.05.

^g^ Pearson's *χ* ^2^ test result for difference between farmer/rancher and nonfarmer/rancher age 65 and older is significant at *p* < 0.05.

*Source*: CDC National Violent Death Reporting System data from reporting states (2003–2018).

**Table 4:** Demographic characteristics of suicide decedents by age, 40 states,^a^ 2003–2018

	Age 18–45			Age 46–64			Age 65 and over		
Mechanism	Farmer/rancher, *N* (%)	Nonfarmer/rancher, *N* (%)	aOR^b^ (95% CI)	Farmer/rancher, *N* (%)	Nonfarmer/rancher, *N* (%)	aOR^c^ (95% CI)	Farmer/rancher, *N* (%)	Nonfarmer/rancher, *N *(%)	aOR^d^ (95% CI)
Firearm^e^^,^ f ^,^ g	284 (62.4)	46,780 (44.4)	1.9*** (1.6–2.3)	441 (71.2)	39,336 (48.8)	2.1*** (1.8–2.6)	711 (82.6)	26,066 (70.0)	1.5*** (1.3–1.9)
Hanging^e^^,^ f ^,^ g	114 (25.1)	35,656 (33.8)	0.6*** (0.5–0.8)	108 (17.5)	17,841 (22.2)	0.7*** (0.6–0.9)	90 (10.5)	4193 (11.3)	1.0 (0.8–1.2)
Poisoning^e^^,^ f ^,^ g	29 (6.4)	14,222 (13.5)	0.6*** (0.4–0.8)	48 (7.8)	16,497 (20.5)	0.5*** (0.3–0.6)	31 (3.6)	4500 (12.1)	0.5*** (0.3–0.7)
Other^e^^,^ f ^,^ g	28 (6.2)	8761 (8.3)	0.7 (0.5–1.1)	22 (3.6)	6882 (8.5)	0.4*** (0.3–0.7)	29 (3.4)	2479 (6.7)	0.6*** (0.4–0.8)
Event history									
History of suicidal thoughts^f^^,^ g	89 (19.8)	21,631 (20.5)	1.0 (0.8–1.2)	95 (15.4)	15,204 (18.9)	0.8** (0.6–1.0)	113 (13.1)	6951 (18.7)	0.7*** (0.6–0.8)
History of suicide attempts^e^^,^ f ^,^ g	59 (13.0)	22,281 (21.1)	0.6*** (0.5–0.8)	62 (10.0)	14,771 (18.3)	0.6*** (0.5–0.8)	28 (3.3)	3491 (9.4)	0.5*** (0.3–0.7)
Life stressors									
Financial problems^g^	37 (8.1)	8885 (8.4)	0.9 (0.6–1.3)	93 (15.0)	10,449 (13.0)	1.1 (0.9–1.4)	32 (3.7)	2025 (5.4)	0.8 (0.6–1.2)
Physical health problems	40 (8.8)	8252 (7.8)	1.2 (0.8–1.6)	128 (20.7)	17,618 (21.9)	0.9 (0.7–1.1)	463 (53.8)	18,807 (50.5)	0.9 (0.8–1.1)
Job problems^f^	41 (9.0)	11,808 (11.2)	0.7** (0.5–1.0)	58 (9.4)	10,882 (13.5)	0.6*** (0.5–0.8)	12 (1.4)	760 (2.0)	NSR
Death of a friend or family	25 (5.5)	4507 (4.3)	1.3 (0.9–2.0)	40 (6.5)	5165 (6.4)	1.1 (0.8–1.5)	78 (9.1)	3424 (9.2)	0.9 (0.7–1.1)
Recent criminal legal problem	52 (11.4)	11,495 (10.9)	1.0 (0.7–1.3)	52 (8.4)	5939 (7.4)	1.0 (0.8–1.4)	12 (1.4)	840 (2.3)	NSR
Recent suicide of friend or family	10 (2.2)	2225 (2.1)	NSR	11 (1.8)	1414 (1.8)	NSR	14 (1.6)	521 (1.4)	NSR
Eviction or loss of home^e^^,^ ^f^	s	2647 (2.5)	NSR	11 (1.8)	3255 (4.0)	NSR	19 (2.2)	752 (2.0)	NSR
Interpersonal									
Intimate partner problem^f^^,^ ^g^	149 (32.8)	37,556 (35.6)	0.9 (0.7–1.1)	117 (18.9)	18,247 (22.7)	0.8** (0.6–1.0)	54 (6.3)	3164 (8.5)	0.8 (0.6–1.0)
Family relationship problem^g^	39 (8.6)	7637 (7.2)	1.2 (0.9–1.7)	37 (6.0)	5230 (6.5)	1.0 (0.7–1.4)	21 (2.4)	1527 (4.1)	0.7* (0.4–1.1)
Argument or conflict^g^	58 (12.8)	14,779 (14.0)	0.9 (0.7–1.2)	39 (6.3)	6522 (8.1)	0.8 (0.6–1.1)	21 (2.4)	1580 (4.2)	0.6** (0.4–1.0)
Mental health/substance use									
Mental health problems^e^^,^ ^f^ ^,^ ^g^	169 (37.1)	44,673 (42.4)	0.9 (0.7–1.1)	207 (33.4)	37,486 (46.5)	0.7*** (0.6–0.8)	237 (27.5)	13,070 (35.1)	0.9* (0.8–1.0)
Received mental health treatment^e^^,^ ^f^ ^,^ ^g^	97 (21.3)	27,598 (26.2)	0.8 (0.7–1.1)	129 (20.8)	24,720 (30.7)	0.7*** (0.6–0.8)	141 (16.4)	7852 (21.1)	0.9 (0.8–1.1)
Depressed mood	153 (33.6)	34,883 (33.1)	1.0 (0.8–1.2)	222 (35.9)	29,461 (36.6)	1.0 (0.8–1.2)	314 (36.5)	13,217 (35.5)	1.0 (0.9–1.2)
Alcohol problem^f^^,^ ^g^	87 (19.1)	18,350 (17.4)	1.0 (0.8–1.2)	102 (16.5)	16,188 (20.1)	0.7*** (0.6–0.9)	28 (3.3)	2697 (7.2)	0.5*** (0.4–0.8)
Substance abuse problem^e^^,^ f ^,^ g	72 (15.8)	2052 (20.9)	0.7*** (0.6–0.9)	38 (6.1)	9817 (12.2)	0.5*** (0.4–0.7)	9 (1.1)	966 (2.6)	NSR

*Note*: Age 18–45: Farmer *N* = 455, nonfarmer *N* = 105,419; age 46–64: Farmer *N* = 619, nonfarmer *N* = 80,556; age 65p: Farmer *N* = 861, nonfarmer *N* = 37,238

s Indicates that the value had to be suppressed, due to a low cell count.

Abbreviations: aOR, adjusted odds ratio; CI, confidence interval.

^a^ Alabama, Alaska, Arizona, California, Colorado, Connecticut, Delaware, Georgia, Hawaii, Illinois, Indiana, Iowa, Kansas, Kentucky, Louisiana, Maine, Maryland, Massachusetts, Michigan, Minnesota, Missouri, Nebraska, Nevada, New Hampshire, New Jersey, New Mexico, New York, North Carolina, Ohio, Oklahoma, Oregon, Pennsylvania, Rhode Island, South Carolina, Utah, Vermont, Virginia, Washington, West Virginia, and Wisconsin (not all states are fully reporting).

^b^ Adjusted odds ratios measure the association between the decedent having the demographic characteristic and being a farmer/rancher aged 18–45. The adjusted odds ratio used nonfarmer age 18–45 as the reference group and controlled for race/ethnicity and sex. Therefore, odd ratios for age, race/ethnicity, and sex are not presented. Adjusted odds ratio level of statistical significance is indicated at *p < 0.10, **p < 0.05, ***p < 0.01. NSR = not statistically reliable. Statistical reliability criteria for logistic regression not met because cell frequencies were less than the required minimum.

^c^ Adjusted odds ratios measure the association between the decedent having the demographic characteristic and being a farmer/rancher aged 46–64. The adjusted odds ratio used nonfarmer age 46–64 as the reference group and controlled for race/ethnicity and sex. Thus, odds ratios for age, race/ethnicity, and sex are not presented. Adjusted odds ratio level of statistical significance is indicated at *p < 0.10, **p < 0.05, ***p < 0.01. NSR = not statistically reliable. Statistical reliability criteria for logistic regression not met because cell frequencies were less than the required minimum.

^d^ Adjusted odds ratios measure the association between the decedent having the demographic characteristic and being a farmer/rancher aged 65 and older. Each adjusted odds ratio used nonfarmer age 65 and older as the reference group and controlled for race/ethnicity and sex. Thus, odds ratios for age, race/ethnicity, and sex are not presented. Adjusted odds ratio level of statistical significance is indicated at *p < 0.10, **p < 0.05, ***  < 0.01. NSR = not statistically reliable. Statistical reliability criteria for logistic regression not met because cell frequencies were less than the required minimum.

^e^ Pearson's *χ* ^2^ test result for difference between farmer/rancher and nonfarmer/rancher age 18–45 is significant at *p* < 0.05.

^f^ Pearson's *χ* ^2^ test result for difference between farmer/rancher and nonfarmer/rancher age 46–64 is significant at *p* < 0.05.

^g^ Pearson's *χ* ^2^ test result for difference between farmer/rancher and nonfarmer/rancher age 65 and older is significant at *p* < 0.05.

*Source*: CDC National Violent Death Reporting System data from reporting states (2003–2018).

### 3.1 Farmers and ranchers compared to nonfarmers

The farmer and rancher suicide decedents were, on average, 60 years old whereas the nonfarmers were, on average, 47 years old (Table 1). While the majority of nonfarmer suicide decedents tend to be younger than 65, 45% of the farmers and ranchers in the data are over 65 years of age. Of farmer and rancher suicide decedents, 91% are non‐Hispanic White males compared to roughly 84% of the nonfarmer suicide decedents. Farmer and rancher suicide decedents were less likely than nonfarmer suicide decedents to be military veterans (aOR = 0.5) even though 22% were military veterans compared to 19% of the nonfarmer suicide decedents.

When controlling for age, race/ethnicity, and sex, farmer/rancher suicide decedents are more likely than non‐farmers to have a high school diploma (aOR = 1.1) or less than a high school diploma (aOR = 2.5) and less likely to have some college or a college degree. Farmers and ranchers who died by suicide are more likely than nonfarmers to live in micropolitan, rural noncore counties adjacent to metro and rural noncore counties nonadjacent to metro (aOR = 2.3, aOR = 4.8, aOR = 3.8, respectively) and less likely to live in large or small metropolitan counties (aOR = 0.2, aOR = 0.7, respectively).

In Table 2, farmer and rancher suicide decedents are more likely to use firearms (aOR = 1.9) than nonfarmers. Firearms were used in 74% of farmer and rancher suicides and 50% in nonfarmer suicides. Farmer and rancher suicide decedents are less likely that nonfarmers to have a history of suicidal thoughts (aOR = 0.8) and a history of suicide attempts (aOR = 0.6). In addition, farmer and rancher suicide decedents are less likely than nonfarmers to have life stressors, interpersonal problems, and mental health/substance use issues as suicide precipitants despite having higher percentages of physical health problems (33% compared to 20%) and experiencing a death of a friend or family (7% compared to 6%)

### 3.2 Female farmers and ranchers compared to male farmers and ranchers

Female farmer and rancher suicide decedents are, on average, younger than male farmer and rancher suicide decedents (53 years old compared to 61 years old). Female farmer and rancher suicide decedents appear to be more educated than male farmer suicide decedents, although the aORs were not statistically reliable. Female farmer and rancher suicide decedents are more likely than male farmer and rancher suicide decedents to live in small metropolitan counties (aOR = 1.9). In Table 2, female farmer and rancher decedents were less likely than male farmer and rancher decedents to use a firearm (aOR = 0.3) and more likely to hang oneself (aOR = 1.7). Female farmer and rancher suicide decedents are more likely to have a history of suicidal thoughts (aOR = 1.6) and a history of suicidal attempts (aOR = 3.0) compared to male farmer suicide decedents.

### 3.3 Farmers and ranchers by age groups

Most of the farmer and rancher suicide decedents were of White race and most were over the age of 65 (Table 3). More farmers and ranchers of American Indian and Alaska Native race or Hispanic/LatinX ethnicity between 18 and 45 died by suicide than farmers of died by suicide than in the other age groups in the other age groups (Table 3). Farmers and ranchers in the 46–64 and 65 and older age groups are more likely than nonfarmers to have educational attainment of less than a high school diploma (aOR = 2.0 and aOR = 3.1, respectively). While farmer and rancher suicide decedents are more likely to live in rural areas, those aged 46–64 are over five times more likely than nonfarmers to die by suicide in rural noncore (adjacent and nonadjacent to metro) counties (aOR = 5.8 and aOR = 5.1). Within most of the age groups, farmer suicide decedent incident characteristics are not statistically different from nonfarmers (Table 4). Variation exists between farmer suicide decedent age groups in counts (*N*) and percentages. A higher count and percentage of farmers between 46 and 64 years old had financial problems (15%) or job problems (9%) compared to the other farmer age groups. Farmer suicide decedents age 65 and older had higher count and percentages of physical health problems (54%), death of a friend or family (9%), and eviction/loss of home (2%) than the other farmer age groups. In addition, a higher count and percentage of young farmer suicide decedents (age 18–45) had intimate partner problems (33%) and arguments/conflicts (13%) before the event.

## 4 DISCUSSION

This analysis of farmer and rancher suicide deaths from 40 states reporting to the NVRDS from 2003 to 2018 identified 1935 deaths by suicide. Of these 1935 deaths, 91% occurred among male, non‐Hispanic, White farmers, and ranchers. Recent analyses of the NVDRS describing farm‐related suicides yielded more events, 2106^22^ incidents and 2801^23^ despite the more narrow study periods, 2003–2018 and 2003–2016, respectively.^22^, ^23^ The discrepancies in incidents are likely due to differences in defining *farmers* and *ranchers*. Our analysis was limited to suicide incidents among *Farmers, ranchers, and other agricultural managers* (Census Code 0205) within *Crop production* (Census Code 0170) or *Animal production* (Census Code 0180), as classified by the Census classification system, whereas other analyses included agricultural workers, laborers, and other nonagricultural production occupations.

Our results are consistent with results of suicide among the general population and agricultural population. Among the general population in the United States, male farmers and ranchers are over three times more likely to die by suicide than females, despite females being more likely to have a suicide attempt.^27^, ^30^ Analyses of segments of the agricultural population have also found males represent a large proportion of identified suicides.^20^, ^22^, ^23^, ^31^ The proportion of suicides among males may be due to several factors. First, this may be reflective of the make‐up of the agricultural workforce, which remains largely male. It may also be related to stigma, in that female producers are more willing to seek help, which resulted in suicide prevention.^32^ Despite 95% of suicides among farmers and ranchers occurring among males, the number of female producers is increasing,^33^ and suicide prevention among females should be a public health priority. Female farmer and rancher suicide decedents were significantly more likely to have experienced suicidal thoughts than men, as reported by an acquaintance, and more likely to have had a history of suicide attempts, both which may offer opportunity for intervention. In addition, female farmer and rancher suicide decedents tended to be younger (age 18–45 and 46–64) and significantly more likely to have resided in a small metropolitan area. These results may describe female farmers and ranchers who engage in small‐scale, urban, or suburban agriculture. In the United States, female producers are slightly younger than male producers and more likely to be beginning farmers and ranchers and more likely to report farm sales less than $50,000.^33^ We did not observe differences in suicide risk by life stressors, interpersonal conditions, or mental health/substance use history, however, female operators have reported work‐related stressors including sexism, stereotypes, and invisible work—unpaid labor—^34^ which are not reported in the NVDRS data set but may contribute to suicide risk. Future research examining differences in risk factors by sex of farmers and ranchers could inform sex‐specific programming and interventions. Interventions for small‐scale and/or urban and suburban producers at relevant conferences and through respected grower organizations could be important to preventing suicide among female farmers and ranchers.

In the US nonfarm population, those aged 45–54 years and 55–64 years consistently have the highest rate of suicides across age groups, ranging from 19.7 to 20.4 and 18.3–18.9 per 100,000 population, respectively.^27^, ^30^ When we consider deaths by suicide among farmers and ranchers 65 years of age and older, occupational characteristics alone may not be risk factors and suicide explanation may be more complex. Risk factors for suicide among older nonfarm include social disconnectedness, bereavement, neurocognitive impairments, and decision making, as well as chronic illness or disability.^35^, ^36^ Less than half (48%) of farmer and rancher suicide decedents aged 65 and older were married, suggesting social disconnectedness and/or loss of spouse or bereavement may have contributed to suicidal action. Furthermore, over half of the farmer and rancher suicide decedents (54%) were experiencing a physical health problem at the time of their death, which may have impacted their overall quality of life or ability to contribute to work on the farm or ranch. For many agricultural producers, farming or ranching is a major construct of their identify and loss of identity due to retirement, by choice or forced, may contribute to suicide ideation.^37^ In a cross‐sectional study of Midwestern farmers, *coping with self‐blame* was the only characteristic, among various psychological, social, and contextual variables, that was significantly associated with self‐reported suicide risk.^38^ Anecdotally and theoretically, economic loss, bankruptcy, or other financial disasters contributing to a sense of failure, blame, and loss of identity have preceded suicide, especially when discussing farmers and ranchers aged 65 and over.^37^

Farmer and rancher suicide decedents between 18 and 45 years of age were significantly less likely to have been divorced and a veteran, common risk factors for suicide.^39^ There were no significant differences among farmers and nonfarmers aged 18–45 by life stressors, interpersonal conditions, or mental health conditions. Farmers and ranchers of all ages were less likely to have had a known substance use problem, however, reports of mental health or substance use challenges are based on reports from acquaintances and collected after the death. Farmers and ranchers may not have disclosed their mental health history or due to stigma, which remains pervasive in rural and agricultural communities.^40^ Suicide among younger farmers and ranchers (aged 18–45) should be of concern. Programs to reduce stigma and encourage early intervention should be implemented at young farmer and rancher events. Agricultural‐specific call and text lines should also be advertised and available to help younger farmers and ranchers.

Suicides occur at a higher rate in rural areas compared to urban areas.^41^ Commonly cited risk factors include geographic isolation, access to lethal means, lack of access to mental health care and intervention, and rural ideologies.^22^, ^41^, ^42^ However, our results also suggest farmers and ranchers who died by suicide, specifically females, were also more likely to have resided in micropolitan areas, defined as having urban areas with a population of at least 10,000 but less than 50,000.^29^ These areas may offer more access to mental health care and suicide ideation intervention; however, our results indicate that farmers and ranchers in these areas are still at increased risk of suicide. Farm and ranch suicide prevention efforts should also target less‐rural areas, especially as urban agriculture becomes more common.

Firearms were used in nearly 75% of farmer and rancher suicides, followed by hanging (16%) and poisoning (5%). In the United States, firearms are estimated to be used in over half of all suicides.^43^ Greater firearm availability and access is associated with higher firearm suicide rates^43^, ^44^ and outside of the United States, research shows reducing firearm ownership lowers firearm suicide rates.^45^, ^46^ However, discussion around limiting access to firearms in the United States may be met with resistance especially in rural communities where gun ownership is common and the right to possess a gun may be considered essential to their sense of freedom.^47^ As such, suicide prevention efforts that focus on reducing access and availability to firearms will likely be met with some resistance. Instead, focusing efforts on possible engineering or administrative interventions to gun access in homes may help to reduce firearm suicides in the United States.

In a 2019 analysis of the NVDRS, 49% of suicide decedents had a current diagnosed mental health problem and 37% experienced depressed mood before their death.^27^ These results are higher than the proportion of farmers and ranchers in our sample who had a current diagnosed mental health problem (32%) and experience depressed mood (36%) before their death. Depression and substance use disorders are common diagnoses among suicide decedents, however, lack specificity as predictors of suicide, suggesting suicide risk is more than mental illness. For example, in a sample of nearly 600 Midwestern agricultural producers, symptoms of anxiety and depression were not significantly associated with suicide risk.^38^ Furthermore, just over 15% of suicide decedents in our sample had a history of suicide thoughts, and even less (8%) had a history of a suicide attempt. Suicide prevention efforts often depend on the disclosure of suicidal thoughts, which are considered an early step in the suicidal process. Results from our analysis suggest disclosing suicide ideation, disclosing symptoms of mental health conditions, such as depression, and even a formal diagnosis may not be sufficient to prevent suicides among farmers and ranchers.

Agriculture is recognized as a stressful occupational industry, characterized by extreme environmental conditions, fluctuating markets, high production costs, high debt loads, and interpersonal, family conflict.^15^, ^16^, ^17^, ^19^ Despite this, less than 10% of farmers and ranchers had a known financial problem (8%), job problem (6%), or family relationship problem (5%) that may have been a precipitating circumstance to suicide. Concerns for farm‐related suicide are often heightened when the farm economy experiences financial strain. In 1991, after the farm crisis of the 1980s, an analysis of suicides in 15 states between 1980 and 1985 resulted in a significant association between the declining farm economy and farm suicides.^46^ However, our results do not offer empirical support for this relationship.

Based on our results, the most prevalent precipitating circumstance among farmers and ranchers was physical health problems (33%). Farmers experience high rates of arthritis, musculoskeletal conditions, cardiovascular diseases, skin cancer, hearing loss, and amputations and are at high risk for work‐related injuries, which may result in permanent disability.^48^ Ahmedani et al.^49^ studied 2670 individuals who died by suicide and found physical health conditions including back pain, chronic obstructive pulmonary disease, migraine, and cancer were associated with increased risk of suicide after adjusting for age, sex, mental health, and substance use diagnoses; the suicide risk increased as the number of conditions increased. Suicide prevention strategies among farmers and ranchers should expand beyond increasing farm financial decision making, resilience, and interpersonal conflict resolution to address managing chronic illnesses and increasing quality of life. Additional research into the life experience of farmers and ranchers managing chronic illness and/or permanent limitation could inform programming and intervention strategies.

### 4.1 Strengths and limitations

A consistent limitation to identifying and categorizing farm‐related suicide deaths is correctly identifying farmers and ranchers. Since 2018, the NIOSH added standardized industry and occupation coding for the NVDRS. This improves the quality of the data set for analysis of suicides by occupation. This analysis was limited to individuals identified as *Farmers, ranchers, and other agricultural managers* (Census Code 0205) within the following industries *Crop production* (Census Code 0170) or *Animal production* (Census Code 0180) and did not include ancillary agricultural occupations, such as agribusinesses, farm laborer, or farmworkers, providing results specific to decision makers and operators on farms and ranches. Additionally, this is the first analysis of farmer and rancher suicide decedents, to our knowledge, that includes 2018 NVDRS data.

Despite strengths to this analysis, there are several limitations to acknowledge. NVDRS data are only available from 40 states and therefore our results are not nationally representative. There are also several limitations related to the availability and completeness of data. NVDRS incident data might be limited or incomplete for areas where data‐sharing relationships are not developed fully between NVDRS programs and state health departments, coroners/medical examiners, and law enforcement. Additionally, medical and mental health information are captured from coroner/medical examiner reports and from family members or friends, as opposed to medical reports, which may be limited by the knowledge of the informant (family members, friends).^27^ The study relies on other agencies coding of qualitative interviews and classifying occupation/industries.

There are conditions that may result in an under‐or‐overcount of suicides among farmers and ranchers. Errors in reporting an incident as suicide may result in underreporting of suicide among farmers and ranchers. Incidents may not have been captured by the NVDRS due to stigma of suicide in some communities, which is especially pervasive in rural areas,^41^, ^42^, ^50^ errors by coroners or medical examiners, or errors by abstractors.^19^ In addition, for many variables included in the NVDRS data set, the coding is yes/no with unknown and missing data coded as “no” when they should be coded as unknown or missing. This gives the researcher a false sense that information is known about a decedent when that might not be the case and could affect the reporting of the truth. In addition, many farmers work a full‐time off‐farm job in addition to managing the farm. NVDRS only provides one occupation and depending on the incident situation, who was interviewed, and what occupation was provided, the off‐farm occupation may have been coded instead of the occupation of crop or animal producer (farmer or rancher).

In conclusion, the results of this analysis suggest suicides among farmers and ranchers remain a public health concern. While data on suicide among female farmers and ranchers are suppressed due to low cell counts, research and intervention focused on suicides among female farmers and ranchers is warranted. While no clear risk factor for suicide among farmers and ranchers emerged, the results underscore the complex nature of suicide and the need for multifaceted, culturally competent interventions and campaigns that address suicide risk and prevention at the individual and community levels.

## AUTHOR CONTRIBUTIONS

Cristina Miller and Josie Rudolphi conceptualized the work. Cristina Miller acquired and analyzed the data, Cristina Miller and Josie Rudolphi drafted and revised the manuscript and approved the manuscript for submission. Cristina Miller and Josie Rudolphi agree to be accountable for all aspects of the work in ensuring that questions related to the accuracy or integrity of any part of the work are appropriately investigated and resolved.

## CONFLICT OF INTEREST

The authors declare that there are no conflict of interest.

## DISCLOSURE BY AJIM EDITOR OF RECORD

John Meyer declares that he has no conflict of interest in the review and publication decision regarding this article.

## ETHICS APPROVAL AND INFORMED CONSENT

Centers for Disease Control and Prevention does not require Institutional Review Board approval to analyze the deidentified National Violent Death Reporting System data set.

## DISCLAIMER

The findings and conclusions in this paper are those of the authors and should not be construed to represent any official United States Department of Agriculture or U.S. Government determination or policy.

## Supporting information

Supporting information.

## Data Availability

The data that support the findings of this study are available from Center for Disease Control and Prevention (CDC). Restrictions apply to the availability of these data, which were used under license for this study. Data are available from the author(s) with the permission of CDC.
