# Supplementary material for: Characteristics of suicide among farmers and ranchers: Using the CDC NVDRS 2003–2018
Source: Am J Ind Med. 2022 Jun 7;65(8):675–89. doi: 10.1002/ajim.23399 (PMC9541098; doi:10.1002/ajim.23399)
Supplement: Supplementary file 1 — Supporting information. [file AJIM-65-675-s001.docx]

**Supplemental Materials**

**Table S1: Description of NVDRS Variables**
